# Sensitivity of density-dependent threshold to species composition in arthropod aggregates

**DOI:** 10.1038/srep32576

**Published:** 2016-08-31

**Authors:** Broly Pierre, Ectors Quentin, Decuyper Geoffrey, Stamatios C. Nicolis, Deneubourg Jean-Louis

**Affiliations:** 1 Unit of Social Ecology, Université Libre de Bruxelles, Campus de la Plaine, 1050 Bruxelles, Belgium

## Abstract

How mixed-species groups perform collective behaviours provides unique insights into the mechanisms that drive social interactions. Herein, we followed the aggregation process of two isopod species under monospecific and heterospecific conditions at three population densities. Our experimental results show that the formation of both the monospecific and heterospecific groups responds to a similar threshold function. Furthermore, the two species contribute equally to the mixed-species aggregate growth and are not spatiotemporally segregated. However, we show that the cohesion is weaker and the probability of forming aggregations is lower in heterospecific groups than in monospecific populations. Thus, our results show that amplification processes are shared between species, but that the weighting given to conspecific and heterospecific information differs. We develop a theoretical model to test this hypothesis. The model reproduces our experimental data and shows that a relatively low level of inter-attractions between species is able to generate mixed-species aggregates. Moreover the greater the total population, the lower this parameter value is needed to observe aggregation in both species. This highlights the importance to study not only qualitatively but also quantitatively the heterospecific interactions in mixed-species groups. Finally, the patterns observed could be biologically relevant in favouring the association between species.

In social animals, decision making to carry out collective tasks is mediated by social information and cooperation^1^,^2^. Social information can be based on intentionally communicated information (signals) or on cues that individuals provide inadvertently (public information)^1^,^3^,^4^. Regardless of its origin, social information increases the information available to individuals that are faced with environmental alternatives and can improve the assessment of the quality of environmental patches. Social information induces behavioural changes that may lead to collective processes such as aggregation^5^,^6^,^7^,^8^, one of the most widespread processes encountered in nature due to its adaptiveness for foraging or reducing predation^9^. While many species aggregate at high quality resources or safe locations, it is also common to observe aggregations in homogeneous environments. These are likely to result only from attraction among individuals, i.e., a gregarious tendency.

In the majority of the studies reported so far, the studied individuals are considered as physiologically and behaviourally similar. In fact the interactions between individuals may vary according to sex, age, size, physiology state or strain (e.g. refs 10, 11, 12, 13, 14, 15, 16, 17). An extreme case of inter-individual difference is mixed-species associations. These associations are frequently observed in mammals (e.g. refs 18,19), birds (e.g. refs 20,21), fishes (e.g., refs 22,23), and arthropods (e.g., refs 24, 25, 26), attesting that heterospecific collective processes can occur^4^,^27^. However, the literature mainly addresses the functionality of such associations. For example, individuals adopting similar behaviour in groups composed of non-ecologically identical species can lead to decreased competition for resources (e.g., during foraging) while at the same time maintaining the benefits of large groups (e.g., as an anti-predation strategy) for the different species^9^,^14^,^28^,^29^. Despite experimental investigations, on heterospecific communication (e.g., kairomones^30^) and the existence of a theoretical framework, many questions remain about the dynamics of mixed-species groups and the nature of interspecific interactions among individuals^27^,^31^,^32^,^33^,^34^,^35^. In particular, the role of the types of individuals and social interactions in the initiation and stability of the mixed-species groups is a central issue^36^.

In many gregarious species, monospecific groups emerge from rules that are based on the amplification of local cues^5^,^7^,^8^,^37^. Accordingly, the greater the number of individuals engaged in a behaviour, the greater the probability that conspecifics will exhibit the same behaviour. At the level of heteropecific groups, one hypothesis is that similar mechanisms of the amplification of local cues are involved. However, the cues shared by the species are not necessarily similar, so the weighting given to conspecific and heterospecific information may vary^37^,^38^. An important issue is therefore the role of this weighting on collective processes such as synchronization and cohesion.

Terrestrial isopods represent an interesting model for the study of basic collective behaviour in animals with low cognitive abilities and simple social relationships. Notably, isopods form a remarkable group of fully terrestrial crustaceans that are particularly abundant in soil ecosystems^40^,^41^. Their cycle of activities is shared with many arthropod and vertebrates species, which includes forming aggregates during the resting phase and solitarily exploring the environment during foraging^42^,^43^. Social interactions in terrestrial isopods are assumed to be based on chemical cues^44^,^45^,^46^,^47^,^48^. In particular, in many terrestrial isopod species, aggregation is a widespread collective behaviour^49^, that is particularly adaptive in reducing individual desiccation^50^,^51^. At the same time, the emergence of aggregation in terrestrial isopods is based on amplification mechanisms and involves a critical number or density of individuals^52^. Similar mechanisms occur in gregarious insects as well as social insects^5^,^6^,^8^. Although many co-occurrences of woodlouse species in micro-habitats have been reported in the field (e.g. refs 53, 54, 55), there have been few investigations regarding the mechanisms facilitating these heterospecific aggreagations^56^.

In this work, we examine heterospecific attraction in two common species of woodlouse. We address the general problem of the dynamic of the formation of mixed-species aggregates in a homogeneous environment and its sensitivity to population composition and density. In particular, we show that the dynamics of aggregation are similar for both species when in monoculture as well as in mixed populations. We test the hypotheses that (1) monospecific and heterospecific groups emerge as a result of similar rules of behavioural amplification but under which (2) heterospecific interactions are quantitatively different from monospecific interactions (conspecific interactions are greater than those that are heterospecific one)^38^.

The experimental and theoretical results of this study validate these two hypotheses; the emergence of both monospecific and heterospecific aggregates are governed by qualitatively similar rules. The threshold function, relating the level of aggregation and the population density, is only quantitatively affected by the species composition, especially for intermediate densities, i.e., close to the threshold value. The model developed from the experimental results shows that strong inter-attraction between the two species is not necessary to generate mixed-species aggregations. Our results suggest that heterospecific association may be adaptive in favouring the association between species when interactions in small populations of a single species are not frequent enough to form monospecific groups during the resting period.

## Material and Methods

Individuals of two common species of terrestrial isopods, *Porcellio scaber* Latreille 1804 (Onisicdea: Crinocheta: Porcellionidae) and *Oniscus asellus* Linnaeus, 1758 (Oniscidea: Crinocheta: Oniscidae) were trapped in urban parks at the Free Brussels University (Belgium) and were maintained together in transparent plastic boxes (170 × 105 × 75 mm). The culture boxes were placed at 21 °C ± 2 °C under the natural photoperiod of the region. Within the boxes, the substratum was maintained at a high relative humidity (R.H. > 80%). Individuals were fed with leaf litter from their original habitat. Individuals less than 5 mm in length were excluded.

For the monospecific aggregation experiments, six treatments were implemented using the two species at three different population sizes: 10 *Porcellio scaber* (n = 15), 10 *Oniscus asellus* (n = 15), 20 *P. scaber* (n = 15), 20 *O. asellus* (n = 15), 40 *P. scaber* (n = 20; reused from^57^) and 40 *O. asellus* (n = 15). For the heterospecific experiments, three mixed-species groups were formed at three population sizes: 5 *P. scaber* + 5 *O. asellus* (n = 15), 10 *P. scaber* + 10 *O. asellus* (n = 15) and 20 *P. scaber* + 20 *O. asellus* (n = 15). For each replicate, the mono- or heterospecific groups of woodlice were introduced into a circular arena of 193 mm diameter. A sheet of white paper covered the bottom of the arena and was changed between each experiment. Individuals were first placed at the centre of the arena in a small and removable enclosure (65 mm in diameter) for 30 s to reduce the excitation inherent to the manipulation. The experiments began with the release of the groups from the centre of the arena (t = 0 s).

The aggregation process of each group was followed for 45 minutes and recorded using a Pentax K-r camera (Pentax Corp., Tokyo, Japan) and a TeckNet® C018 webcam (TeckNet®, Liverpool, UK). Two individuals were considered aggregated when they were in contact for at least 2 minutes (see, e.g. ref. 57). We measured aggregation in two ways. First, the *aggregated fraction* was the fraction of the population aggregated into groups of two or more individuals. The *principal aggregate* was the largest (and often the only) aggregate present at the end of experiments. At the end of 45 minutes, the distance between all individuals was measured using Cartesian coordinates determined by the software *Regressi* and its plugin, *Regavi*. The *interindividual distance* was calculated as the average distance between one individual and the rest of the population. The theoretical interindividual distance (i.e., the distance expected from a null model) was calculated from 1000 simulations of a random distribution of individuals. The *distance to the nearest neighbour* was the smallest distance between one individual and another conspecific or heterospecific individual. This last measure for heterospecific groups was only performed in populations of 10 + 10 individuals in which the recognition of species was accurately possible (low stacking of individuals in the aggregate).

A second index was used as a measure of segregation. Many indexes of segregation are described in the literature. Here, we use an index D (eq. (1)), which is based on the dissimilarity index of Duncan and Duncan^58^ and is bounded between 0 (no segregation) and 0.5 (segregation between both species):


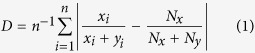


where *x*_i_ (*y*_i_) is the number of woodlice of species X (Y) in cluster *i*. *N*_x_ and *N*_y_ are, respectively, the total populations X and Y in the set-up and *n* is the number of clusters. In our experiments, *N*_*X*_ and *N*_*Y*_ are equal.

The statistical tests and regression analyses were performed using GraphPad Prism 5.01 (GraphPad Software, Inc., La Jolla, California). The kernel density representations were made with Past 2.17. For the normalisation of these representations, the mean angle of each experiment is calculated and normalised as 0°, and the coordinates of individuals are rotated accordingly.

### The model

The model was implemented as a stochastic (multi-agents) simulation based on the present results and previous work on *Porcellio scaber*^52^,^59^,^60^. To aid understanding, we first summarize the main steps of the simulation.

From the empirical observations^52^,^59^,^60^, individuals quickly migrated towards the edge of the arena and walked along its perimeter. The model did not incorporate this aspect, i.e., at time t = 0 s, all the individuals are randomly distributed along the perimeter (600 mm). Consequently, the model is a 1-D simulation with periodic boundary conditions. When the simulation starts, individuals walk clockwise or counter-clockwise along the perimeter (600 mm) of the arena with a mean speed of 1.6 cm.s^−1^ +/− 0.46 cm.s^−1^. The probability of choosing either clockwise or counter-clockwise movement is equal (0.5). The probability of a U-turn is zero except for when an individual leaves a cluster or stops. The model does not incorporate any spontaneous stops, as these were rarely observed during the experiments. The probabilities of stopping (s^−1^) and of leaving and starting to move depend on the population composition. In our analysis, woodlice were considered to be aggregated when two or more individuals were at a distance of less than 8 mm from each other, which corresponds to the length of a woodlouse including its antennae. To be in agreement with experimental data, only individuals at rest are assumed to be aggregated. Only the cases in which the introduced populations of *O. asellus* and *P. scaber* are equal are discussed in this paper. N_x_ and N_y_ represent the total population of each species. The outputs of the simulations are the number of clusters and the number of aggregated woodlice, and thus the aggregated fraction of the total population.

For each set of parameters, 5000 simulations were performed and the results are shown at t = 45 minutes (2700 s).

### Monospecific situations

In this case, the probability of stopping (per unit of time, s^−1^) increases with the number of woodlice in the neighbourhood and is calculated as


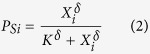


where X_i_ is the number of woodlice in the neighbourhood of individual *i*, i.e., the total population at a distance of less than 8 mm from *i*. This distance represents the range of mutual influence between individuals. The probability of leaving decreases with the total population within the neighbourhood of the individual as


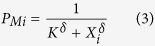


### Heterospecific situations

We take the simplest hypothesis and allow the same mechanisms to govern the dynamics of aggregation. Furthermore, we assume that the response to conspecifics is higher than the response to a heterospecific. In equations (4) and (5) below, the parameter β (0 ≤ β ≤ 1) characterizes the strength of inter-attraction between the two species. We assume that this inter-attraction between species is symmetrical (the influence of species 1 on species 2 is equal to the influence of species 2 on species 1). If β = 0, there is no interspecific influence and if β = 1, there is no discrimination between the species.

The probability of stopping and leaving for individual *i* of species 1:


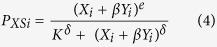



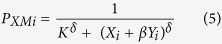


and for individual i of species 2:


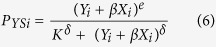



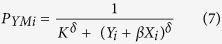


where X_i_ and Y_i_ represent the neighbours of species 1 and 2, respectively.

#### Indexes of aggregation/segregation

In the case of the heterospecific population simulations, we estimate the level of segregation between clusters. The index D (see eq. 1) is calculated at the end of each simulation and is averaged over all simulations. Simulations where no cluster was formed were not taken into account when calculating this mean value.

## Results

### Empirical observations of woodlice aggregation behaviour

After release from the centre of the arena, the individuals quickly migrated towards the edge of the arena (in less than 1 min, 90% of individuals were outside the central area^58^) and, due to a strong thigmotactic behaviour, the population quickly formed a crown along the perimeter of the arena before forming clusters. The mean speed of a woodlouse in the arena was 1.46+/− 0.57 cm.s^−1^ (n = 96 individuals) and the mean probability of making a U-turn (spontaneously or after a crossing with another individual) was 0.0005 per cm (n = 96 individuals).

**Figure 1:**
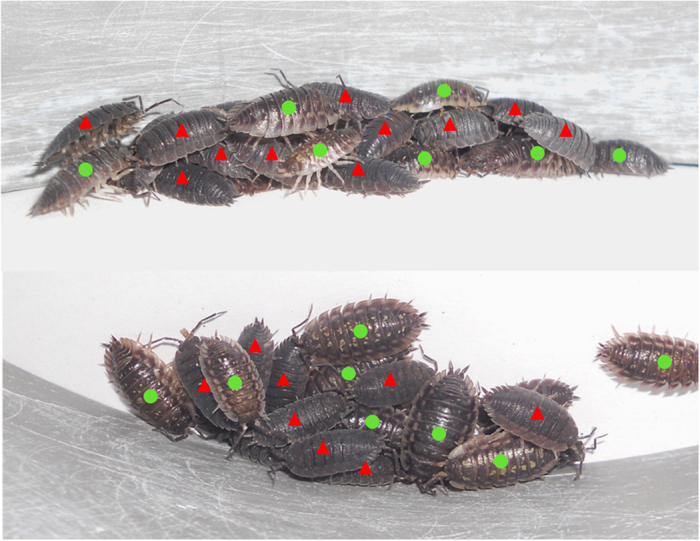
Top and profile view of mixed-species group of woodlice in experiments that included 40 individuals (20 *Porcellio scaber* (triangle) and 20 *Oniscus asellus* (circle)).

**Figure 2:**
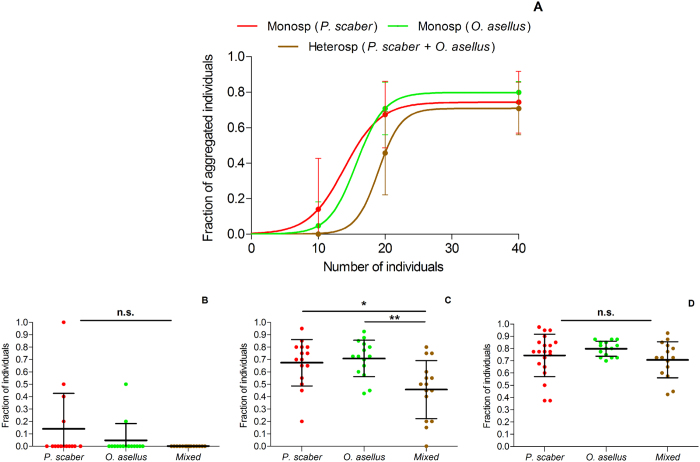
(**A**)Fraction of aggregated individuals at the end of the experiments according to the population size introduced. The solid lines fit the data with a threshold function (only the means are calculated here for better visibility, but the fittings are calculated using all of the data given in 2B–D). The equation is a logistic function 
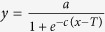
 with a threshold value *T*. For the monospecific groups of *P. scaber*, a = 0.7438, T = 13.93, c = 0.3719 (df = 48; R^2^ = 0.6309); for the monospecific groups of *O. asellus*, a = 0.7983, T = 15.74, c = 0.4842 (df = 43; R^2^ = 0.8967) and for the heterospecific groups, a = 0.7083, T = 19.8, c = 0.5969 (df = 43; R^2^ = 0.7879). Distribution of the fraction of individuals aggregated at the end of the experiments under the different monospecific and heterospecific conditions, which included 10 individuals **(B)**, 20 individuals **(C)** or 40 individuals **(D)**. Bars represent the means and SD for each species composition. *Denotes a significant difference between species composition (see Table 1 for values).

For the three types of species combinations (*P. scaber*, *O. asellus*, mixed groups), the aggregated fraction of the population increased with the total number of tested individuals (10, 20, 40 individuals), from very-weakly aggregated patterns in small populations to highly aggregated patterns in large populations (Figs 1 and 2; Table 1A). The aggregated fraction increased with a similar logistic function for the three species combinations (Fig. 2A). However, there were quantitative differences between the monospecific and heterospecific conditions (comparison of best-fit values with an extra sum-of-square F test of fits, F = 4.392, P = 0.0004; Fig. 2, Table 1B). Indeed, heterospecific groups were more weakly aggregated than monospecific groups, especially in the population of 20 individuals (Fig. 2C; Table 1B). Additionally, the heterospecific experiments including 5 + 5 individuals never presented a stable aggregation, in contrast to the monospecific experiments using similar population sizes (Table 1B, Fig. 2B). There were no species-specific differences in the large populations of 40 individuals (Table 1B; Fig. 2D). The aggregation patterns between the two monospecific conditions were not different, regardless of the density (Table 1B; Fig. 2). The threshold values of the logistic functions for the three species combinations were T = 13.9 for *P. scaber*, T = 15.7 for *O. asellus* and T = 19.0 for the mixed group (Fig. 2A).

**Figure 3:**
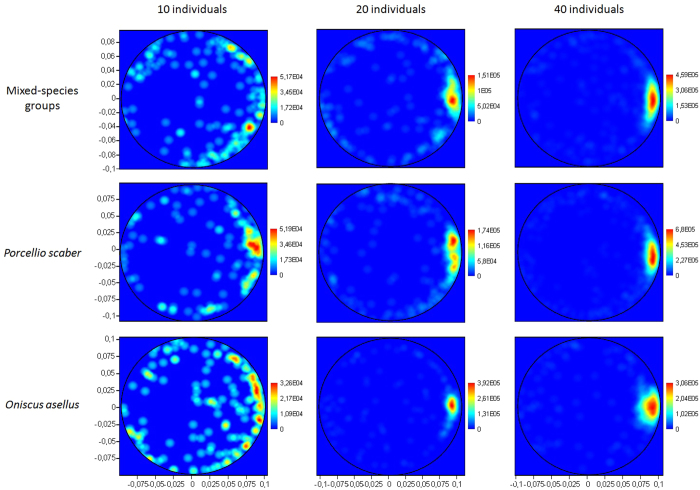
Normalised kernel density representation (paraboloid function) of the mean spatial distribution of the individuals under the 9 tested conditions.

The spatial spreading of the individuals (interindividual distance and distance to nearest neighbour) decreased with group size, regardless of the species composition (Fig. 3; Table 1A; Figs S1 and S2). When aggregation did not occur (i.e., in small populations), the dispersion of individuals was close to a random distribution (≈0.12 m; Fig. 3; Fig. S1). Additionally, heterospecific groups exhibited more dispersed patterns than did monospecific groups (Table 1B).

**Figure 4:**
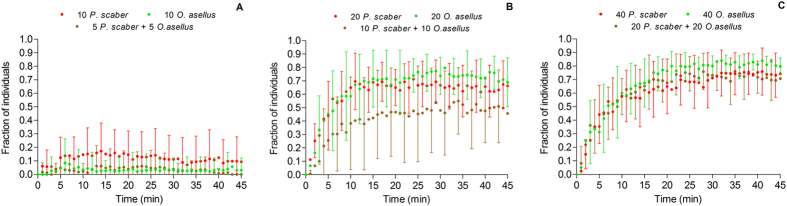
Dynamics of aggregation (fraction of the total number of introduced individuals) of mono- and heterospecific groups in experiments with 10 individuals **(A)**, 20 individuals **(B)** and 40 individuals **(C)**.

When aggregation did not occur (mainly in populations with 10 individuals), the individuals were persistently mobile in the arena. When aggregation occurred (mainly in populations with 20 and 40 individuals), the dynamic of the phenomenon was rapid; in less than 10 minutes, more than 50% of the individuals were aggregated, with the exception of the heterospecific populations that included only 10 + 10 individuals (Fig. 4). After this growth phase, the aggregation was stable and presented a plateau phase (in which the value differed depending on conditions, see above; Fig. 4). Individuals were aggregated in only one aggregate by the end of experiment (no difference between populations of *P. scaber* and *O. asellus*; Fig. S3), even if several aggregates sometimes formed during the first minutes of the experiments (Fig. S3).

**Figure 5:**
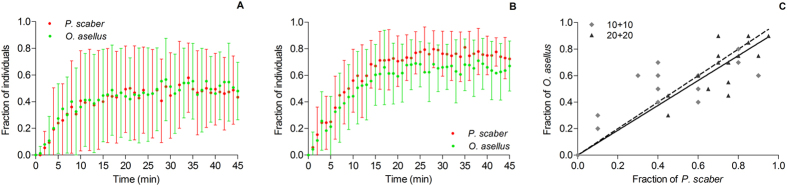
Dynamics of the aggregation of the two species *Porcellio scaber* and *Oniscus asellus* in the principal aggregate in the heterospecific experiments with 20 (10 + 10) individuals **(A)** and 40 (20 + 20) individuals **(B)**. **(C)** Distribution of the aggregated fraction of the two species (at the end of experiments) under the two density conditions. The solid line represents the mean fitting F_Oa_ = 0.939F_Ps_ (df = 29; R^2^ = 0.5205). The dotted line represents the theoretical case F_Oa_ = F_Ps_.

Under heterospecific conditions, the dynamic of the inclusion of the two species in the principal aggregate was similar for the two population sizes at which aggregation occurred (Fig. 5A,B). There was no inter-species difference in the 15 first minutes of aggregate growth (i.e., homogeneity of slopes) in either of the experiments with 10 + 10 individuals (Fig. 5A; one-way ANCOVA, F = 0.0043, P = 0.9481) or the experiments with 20 + 20 individuals (Fig. 5B; one-way ANCOVA, F = 2.719, P = 0.1103). Similar results were obtained for the stability phase of the aggregates from the 15^th^ minute to the end of the experiments (one-way ANCOVA, F = 2.392, P = 0.1276 (Fig. 5A) and F = 0.0696, P = 0.7929 (Fig. 5B), respectively). At the end of the experiments (45^th^ min), there was no significant difference in the mean fraction of *P. scaber* and *O. asellus* aggregated in the principal aggregate for both of the experiments with 10 + 10 individuals (Wilcoxon test, p = 0.3652) and those with 20 + 20 individuals (Wilcoxon test, P = 0.1016). Additionally, the relationship between the aggregated fractions of the two species was close to 1, corresponding to a similar level of aggregation for both species under mixed conditions (Fig. 5C).

Finally, as for the global aggregation, the mean fraction aggregated in the principal aggregate of each species was significantly lower in the experiments that included 10 + 10 individuals than in those with 20 + 20 individuals, (*P. scaber*: Mann-Whitney test, U = 42.0, P = 0.0037; *O. asellus*: Mann-Whitney test, U = 57.0, P = 0.0225).

**Figure 6:**
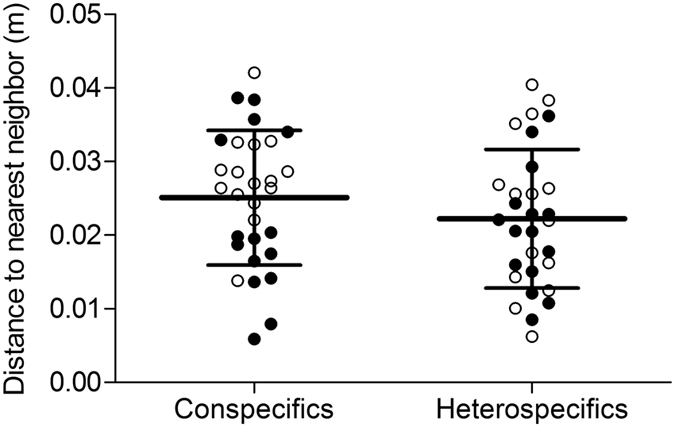
Distributions of the mean distances to the nearest conspecific or heterospecific neighbour in the heterospecific experiments with 20 individuals (at the 45^th^ min). The black points represent *P. scaber* and the white points, *O. asellus*. Bars represent the means and SD for the pooled data.

In heterospecific groups, the distance to the nearest neighbour (regardless of the species of the neighbour) was similar for the two species at the end of the experiments (Mann-Whitney test, U = 48.0, p = 0.9118). Additionally, the individuals were not significantly nearer to conspecific neighbours than they were to those that were heterospecific (Fig. 6; Mann-Whitney test, U = 367.0, P = 0.2226). For 10 + 10 individuals and 20 + 20 individuals, the index of segregation were close to zero (0.1003 +/− 0.0853; 0.0355 +/− 0.0353, respectively) and were significantly different (Mann-Whitney test, U = 59.0, P = 0.0459). For these two conditions, the mean number of clusters per experiment was close to 1 and was not significantly different (0.93 +/− 0.25; 1.13 +/− 0.35, respectively; Mann-Whitney test, U = 91.0, P = 0.0943).

### Theoretical results

**Figure 7:**
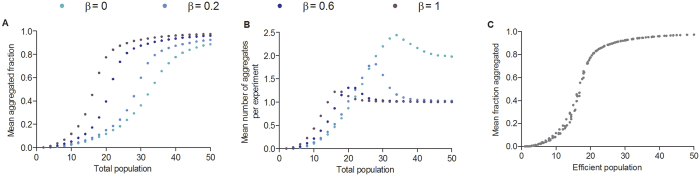
(**A,B)**. Mean fraction of aggregated individuals **(A)** and mean number of clusters per experiment **(B)** at the 45^th^ minute as a function of the total population introduced (modelling results). The results are given for different levels of inter-attractions between individuals β = 0; β = 0.2; β = 0.6 or β = 1 (also equal to the conspecific interaction). Parameter values: K = 2; ε = 2. Two species Nx = Ny = 0.5N, N represents the total population. **(C)** Mean fraction aggregated at the 45^th^ minute as a function of the efficient population 0.5N(1 + β).

In the cases of monospecific (β = 1) and heterospecific simulations, the mean aggregated fraction increased with the total population in a sigmoidal fashion (Fig. 7A). For each β tested, the mean number of clusters per simulation reached a maximum before reaching a plateau at value = 1. For populations greater than 20 individuals, all the individuals were aggregated into one cluster (Fig. 7B). For each value of β, the total population that maximizes the number of clusters was close to the total population for which the aggregated fraction is equal to 0.5 (the threshold values) (Fig. 7A,B). This suggests that the abrupt increase in the aggregated fraction was mainly due to the formation of a large number of small clusters. In addition, we can show that the mean aggregated fraction only depends on a new scale variable, Σ, involving the total number of individuals and the value of β:


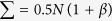


Thus, any combination of N and β for which Σ is constant gives the same mean fraction of the aggregated population (Fig. 7C).

**Figure 8:**
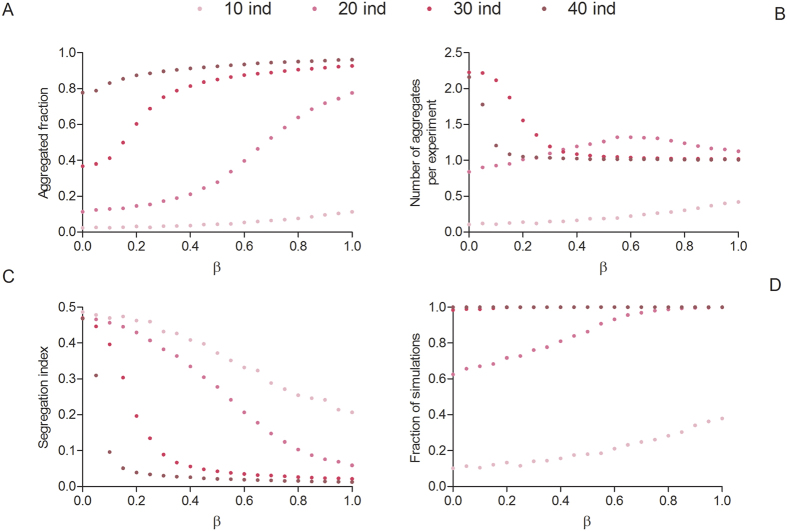
Mean fraction of aggregated individuals **(A)**, mean number of aggregates per experiment **(B)**, segregation index **(C)**, and fraction of simulations with at least one cluster **(D)** as a function of β for different values of the total population (modelling results).

Figure 8 displays the aggregated fraction, the number of aggregates per experiment, the segregation index and the fraction of simulations where at least one aggregate is observed as a function of the inter-attraction parameter β. We note that:

- The fraction of aggregated individuals increases with β in a more or less sigmoidal fashion for intermediate values of *N* (Fig. 8A).
- As β increases, the mean number of clusters increases for a small population (10 individuals) while for the other sizes tested, the mean number of clusters displays a maximum at a particular β value (Fig. 8B).
- The segregation index D (eq. 1) always decreases as a function of β, reaching almost 0 for N ≥ 30 and β > 0.4 (signaling therefore the formation of a unique cluster composed of individuals of both populations) (Fig. 8C). The situation is qualitatively different for β < 0.4, as individuals are aggregated in smaller clusters (Fig. 8A,B) composed of only one species (i.e., the two populations are segregated).
- For a small population size, many simulations end up with no aggregate (Fig. 8D).

The overall results show that aggregation is rather robust, and a small value of interspecific attraction is enough to favour aggregation of both species in the same clusters. In our cases with *P. scaber* and *O. asellus*, the comparison between theoretical and experimental results (mean number of clusters, mean aggregated fraction) suggests that the β value is approximately 0.6.

## Discussion

Which individuals and which social interactions are involved in the collective decision-making process are central to understanding the initiation and stability of groups. Numerous investigations have been devoted to monospecific grouping and the modulation of behavioural rules according to social context^5^,^7^,^8^. In contrast, few studies have considered the interactions involved in the collective behaviours in heterospecific groups^27^,^33^,^34^,^35^,^36^,^39^,^61^, though mixed-species associations have been frequently observed in many vertebrates (e.g. refs 18, 20 and 62) and invertebrates (e.g., refs 24, 25, 26). In this context and in light of the general behavioural rules observed at the species level, we investigated the collective decision-making process and its sensitivity to group size in monospecific and heterospecific aggregations of terrestrial isopods.

### Monospecific aggregation

In terrestrial isopods, as well as in eusocial (e.g., ref. 63) or gregarious insects (e.g. ref. 64), monospecific aggregation is based on the social attraction between individuals and the amplification of these local cues^52^,^57^,^65^. Therefore, the greater the number of conspecifics engaged in a cluster, the greater the attractiveness of the cluster is^5^,^7^,^8^,^37^. The quick formation of only one large aggregate in a homogeneous environment highlights such a social component^5^,^6^,^57^,^64^,^66^. More precisely, in this work, we show that the formation of the aggregate requires a critical number of individuals (critical density), as evidenced by the amplitude of the size of the aggregates in small populations^67^,^68^,^69^. In monospecific populations, the fraction of aggregated individuals is a sigmoidal function of the population size. The sigmoidal function shows an abrupt increase in the aggregated fraction with an increase in total population. Below a critical total population (the threshold), this aggregated fraction is small, and after it reaches a plateau value, most of the individuals are aggregated.

The relation with the mean aggregated fractions and the total population are qualitatively and quantitatively similar (sigmoidal functions) for the two studied species. In both *Oniscus asellus* and *Porcellio scaber*, the low number of individuals necessary to generate an aggregation and the rapid dynamic of the phenomenon are in accordance with the benefit of grouping to reduce individual water loss^50^,^51^. Because *O. asellus* has a higher desiccation rate than *P. scaber*^70^, we expected than *O. asellus* would be more gregarious than *P. scaber*^71^. This expectation was only weakly upheld in our study. However, our experiments on *O. asellus* display a remarkably low variance between each trial, suggesting that small aggregates could be less endurable for this species.

### Heterospecific aggregation

In our study, the woodlice quickly formed mixed-species aggregates. In particular, the relationship between aggregation and population size (sigmoidal function) is qualitatively similar in the monospecific and mixed-species groups (i.e., aggregation increases with population size). In mixed-species groups, the contributions of the two species for the initiation and growth of the aggregates are similar and the aggregation dynamics of the two species are symmetrical. In addition, the spatial distribution of the two species is similar at the group level. There was no spatial-temporal segregation of the two species in the heterospecific aggregates. Together, these results strongly suggest that the two species share similar mechanisms of the emergence of their aggregation patterns and that both species influence and respond to each other. Our model shows that a relatively low level of interactions between species, approximately 50% of the intraspecific inter-attraction, is able to generate mixed-species aggregates. Moreover, for a large population, the aggregation of both species in the same cluster is reached with a low value of inter-attraction between species (e.g., β = 0.2, N ≥ 40). From the comparison between simulations and experimental results, we suggest that the β in our case is approximately 0.6. In this study, we were able to infer the interspecific attraction (β) because the experiments were conducted using different group sizes, mainly small groups. In contrast, we cannot infer β only using large groups because they always form aggregations. This finding supports the idea that the quantification of β models requires the study of group sizes that are close to the critical value necessary for the emergence of aggregation. This finding should be considered in future studies of other heterospecific models.

Mixed-species groups that had a lower number of individuals that was close to the threshold value of the monospecific groups, however, showed a clear reduction in their aggregation patterns (i.e., aggregation probability decreases more quickly with group size than under monospecific conditions). Specifically, mixed-species groups of only 5 + 5 individuals never presented aggregation patterns which differed from what was observed in the monospecific groups of 10 individuals. The theoretical result is in agreement with the experimental results.

The threshold function in self-amplified groups is sensitive to environmental factors and social context (e.g. refs 67,68). A slight disturbance in information transfer may increase the threshold value and therefore decrease the probability of obtaining a stable aggregate, especially in small populations. For example, the threshold in a homogeneous world (such as the present study) appears to be higher than in our previous studies using shelters^52^, most likely because the absence of a favourable aggregation site in the experimental set-up weakens the emergence and stability of aggregates. Therefore, it is not surprising that experiments with small populations that are close to the critical number required to generate an aggregate are more sensitive to the species composition of the group. In this respect, our results are in accordance with the amplification processes being shared between species, but the weighting given to conspecific and heterospecific information is not strictly identical. The results strongly suggest that heterospecific inter-attractions are weaker than monospecific inter-attractions, but we cannot draw any definitive conclusions about individual recognition (whether only one or both species is/are less receptive to heterospecific signals) and the nature of the signal. The cues governing the inter-attraction between conspecifics are most likely tactile and chemical^45^,^48^,^52^. Tactile interactions should not be species-specific and therefore allow minimal heterospecific inter-attraction (β > 0). One hypothesis is that oniscidean species share some, but not all, common molecules that may be involved in heterospecific inter-attraction, as reported in stink bugs^72^ and locusts^73^.

In conclusion, our results suggest that a similar mechanism of amplification should be involved in monospecific and heterospecific groups, thus implying inter-attraction occurs between conspecifics and a similar, but weaker, inter-attraction occurs between heterospecifics. This finding is in agreement with results in mixed-species flocks of foraging birds and aggregation of arthropods^27^,^38^,^39^,^61^,^74^ or, at a more specific level, in different strains of cockroaches^12^,^75^ or families of earwigs^76^. In addition, we showed that a small value of interspecific attraction is enough to form mixed aggregation; a quantification, if it is generalized, that might suggest that such associations could be easily produced in many vertebrates and invertebrates.

However, attraction to heterospecifics is not necessarily always required to produce heterospecific aggregations, as evidenced by the mixed-species colonial breeding that is observed in weaverbirds and is mainly driven by an optimal distribution of conspecific nests^77^. Indeed, in weaverbirds, no evidence for heterospecific attraction was found, but their mixed aggregations instead include a tolerance to heterospecific proximity and a limitation in the number of aggregation sites^77^. Thus, similar mixed patterns can emerge from different networks of interactions among species and their responses to the environment. This reflects the need to study collective decision-making by qualitatively and mainly quantitatively considering the information used by both conspecifics and heterospecifics. One important experimental set-up useful for such explorations of collective decision-making is a homogeneous set-up, like that used in this study, as, such a set-up lacks the possibility of resource competition as a result of heterogeneity such as shelter so that mixed-species aggregation can only result from a minimal heterospecific inter-attraction.

Quantifying heterospecific interactions allows predictions to be made about the distribution of individuals, especially in regard to variations in the habitat structure. In a heterogeneous world with aggregation patches in excess, it is expected that individuals may form mixed-species aggregations even in the case of small heterospecific interaction (β). However, when resources are limited, leading to stronger competition between individuals^78^,^79^, the robustness of a mixed-species association to environmental fragmentation depends on the strength of the heterospecific inter-attractions. We assume that for high value of β, the association between species could be robust but, in contrast, a low value may lead to a segregation of species^61^.

The degree of conspecific aggregation reflects the costs and benefits of living in group^9^. Similarly, heterospecific aggregation may be robust and adaptive if individuals of both species are involved in the same profit objective, including protection from predation, increased foraging efficiency or a reduction in environmental stresses^18^,^19^,^21^. However, the efficiency of the cooperation (at least at the intraspecific level) depends on the size of the group^9^. Independently of the nature of the benefit, our results confirm the assumption that an association between species can occur when the populations of a single species are not sufficient to maintain monospecific group cohesion, thus allowing mixed species aggregations to reach a high efficiency of cooperation. In our study, most of the small monospecific populations did not show aggregation, but when two populations of heterospecifics interacted, aggregations were formed systematically. For woodlice, aggregation to resist against desiccation is important^44^,^45^,^50^,^51^ and heterospecific attraction could promote reciprocal benefits in grouping with other individuals (whether of the same species or not). In other words, for each species, heterospecific aggregation may be more adaptive than a monospecific dispersed distribution.

## Additional Information

**How to cite this article**: Pierre, B. *et al*. Sensitivity of density-dependent threshold to species composition in arthropod aggregates. *Sci. Rep.* **6**, 32576; doi: 10.1038/srep32576 (2016).

## Supplementary Material

Supplementary Information

## Figures and Tables

**Table 1:** A. Statistical results (Kruskal-Wallis test followed by a Dunn’s post-test) of the intra-species comparisons of the aggregated fraction and the interindividual distance (at the end of experiments) among the three group sizes.

	Monospecific		Heterospecific
	*Porcellio scaber*	*Oniscus asellus*	
**A**			
Fraction aggregated	KW = 22.56, p <0.0001 10 ind. < 20 ind. (P < 0.01) and 40 ind. (P < 0.001)	KW = 31.23, p <0.0001 10 ind. < 20 ind. (P < 0.001) and 40 ind. (P < 0.001)	KW = 33.08, p <0.0001 5 + 5 ind. < 10 + 10 ind. (P < 0.001) and 20 + 20 ind. (P < 0.001)
Interindividual distance	KW = 13.76, p = 0.001 10 ind. > 40 ind. (P < 0.001)	KW = 29.23, P < 0.0001 10 ind. > 20 ind. (P < 0.01) and 40 (P < 0.001)	KW = 19.40, P < 0.0001 20 + 20 ind. < 10 + 10 ind. (P < 0.05) and 5 + 5 (P < 0.001)
Distance to nearest neighbour	KW = 29.57, P < 0.0001 10 ind. > 20 ind. (P < 0.05) and 40 ind. (P < 0.001) and 20 ind. > 40 ind. (P < 0.05)	KW = 35.82, P < 0.0001 10 ind. > 20 ind. (P < 0.01) and 40 ind. (P < 0.001) and 20 ind. > 40 ind. (P < 0.05)	KW = 35.66, P < 0.0001 10 ind. > 20 ind. (P < 0.01) and 40 ind. (P < 0.001) and 20 ind. > 40 ind. (P < 0.05)
**B**			
Fraction aggregated	**10 individuals**	**20 individuals**	**40 individuals**
	KW = 4.587, p = 0.1009	KW = 10.22, p = 0.0061 Mixed group < *P. scaber* (P < 0.05) and *O. asellus* (P < 0.01)	KW = 2.870, p = 0.2381
Interindividual distance	KW = 0.5480, p = 0.7603	KW = 5.605, p = 0.0606	KW = 2.373, p = 0.3053
Distance to nearest neighbour	KW = 0.0147, p = 0.9927	KW = 0.7150, p = 0.6994	KW = 2.180, p = 0.3362

**B.** Statistical results (Kruskal-Wallis test followed by a Dunn’s post-test) of the inter-species comparisons of the aggregated fraction and the interindividual distance (at the end of experiments) for each group size.
