# Erratum: Sensitivity of density-dependent threshold to species composition in arthropod aggregates

**DOI:** 10.1038/srep34436

**Published:** 2016-09-30

**Authors:** Pierre Broly, Quentin Ectors, Geoffrey Decuyper, Stamatios C. Nicolis, Jean-Louis Deneubourg

Scientific Reports 6: Article number: 3257610.1038/srep32576; published online: 08 31 2016; updated: 09 30 2016

The original version of this Article contained errors in the spelling of the authors Pierre Broly, Quentin Ectors, Geoffrey Decuyper & Jean-Louis Deneubourg which were incorrectly given as Broly Pierre, Ectors Quentin, Decuyper Geoffrey & Deneubourg Jean-Louis respectively.

The Author Contributions Statement,

Conceived and designed the experiments: B.P. and D.J.-L. Performed the experiments: B.P., E.Q. and D.G. Analysed the data: B.P., Q.E., D.G., C.N.S. and D.J.-L. Wrote the paper: B.P., C.N.S. and D.J.-L.

now reads:

Conceived and designed the experiments: P.B. and J.-L.D. Performed the experiments: P.B., Q.E. and G.D. Analysed the data: P.B., Q.E., G.D., S.C.N. and J.-L.D. Wrote the paper: P.B., S.C.N. and J.-L.D.

In addition, the ‘How to cite this article’ section quoted an incorrect abbreviation for Pierre Broly.

These errors have now been corrected in the PDF and HTML versions of the Article.

