# Supplementary material for: Sensitivity of density-dependent threshold to species composition in arthropod aggregates
Source: Sci Rep. 2016 Aug 31;6:32576. doi: 10.1038/srep32576 (PMC5006165; doi:10.1038/srep32576)
Supplement: Supplementary Information [file srep32576-s1.pdf]

# Supplementary material

## Sensitivity of density-dependent threshold to species composition in arthropod aggregates

Broly Pierre[1,*], Ectors Quentin[1], Decuyper Geoffrey[1], Nicolis Stamatios C.[1], Deneubourg Jean-Louis[1]

[1] Unit of Social Ecology, Université Libre de Bruxelles, Campus de la Plaine, 1050 Bruxelles, Belgium

[*] corresponding author: pierre.broly@gmail.com

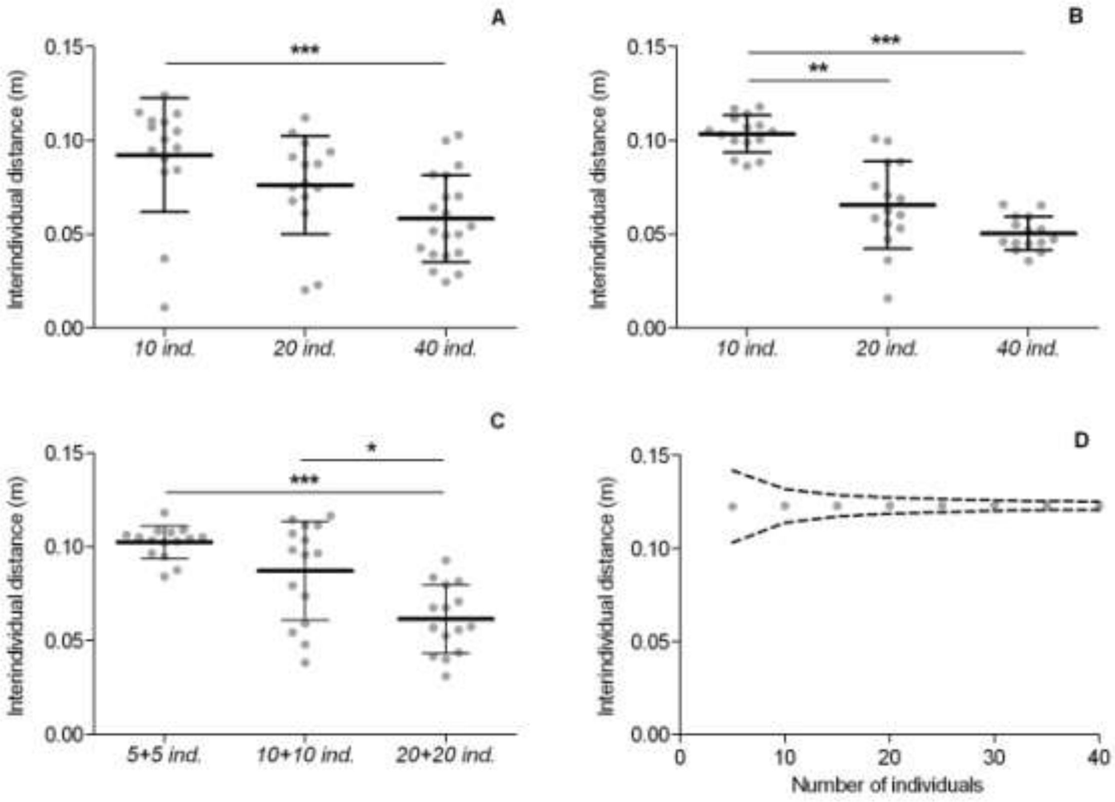


**Figure S1.** For the three group sizes, the distribution of the mean inter-individual distances at the end of the experiments with *Porcellio scaber* (A), *Oniscus asellus* (B) and for mixed groups (C). Bars represent the means and SD for each species composition. * denotes significant difference between group sizes (see Tab. 1 for values). The theoretical relationship between the number of individuals in the arena and their mean (+ SD) interindividual distance in a random distribution is also given (D).

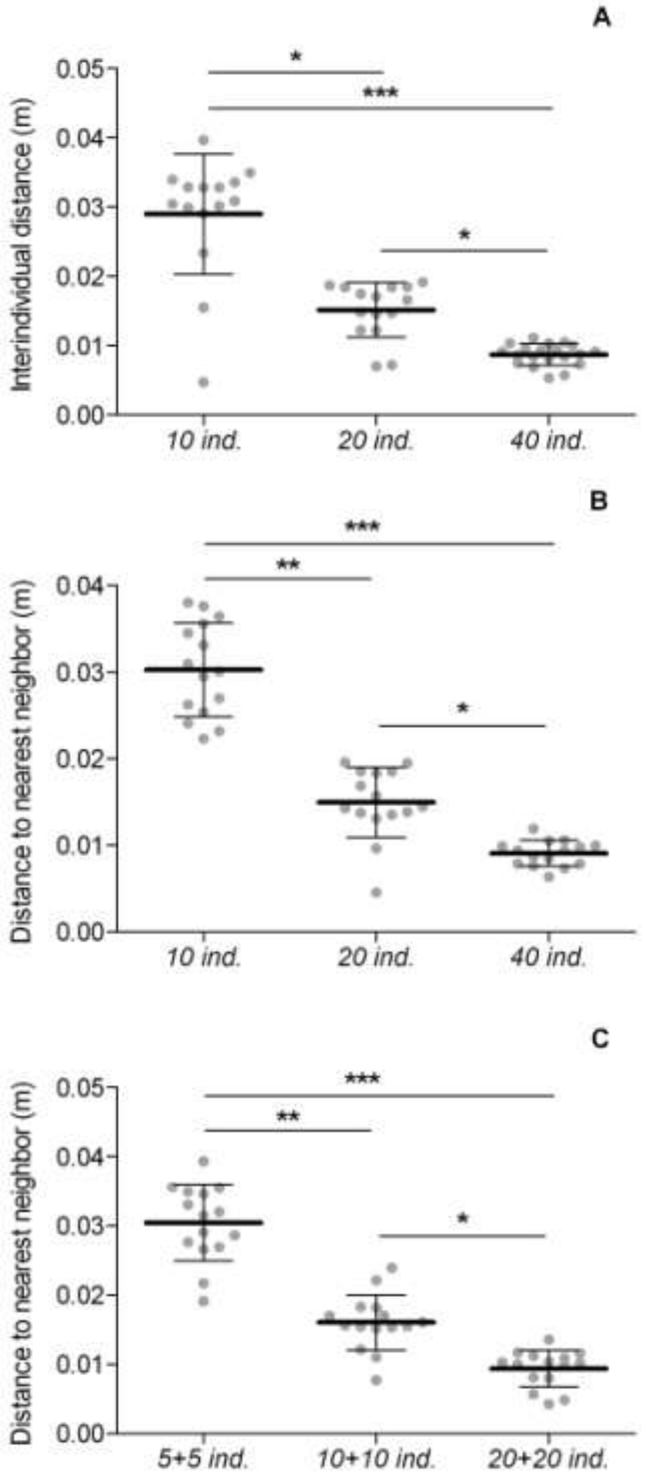


**Figure S2.** For the three group sizes, the distribution of the mean distance to the nearest neighbour at the end of experiments with *Porcellio scaber* (A), *Oniscus asellus* (B) and for mixed groups (C). Bars represent the means and SD for each species composition. * denotes significant difference between group sizes (see Tab. 1 for values).

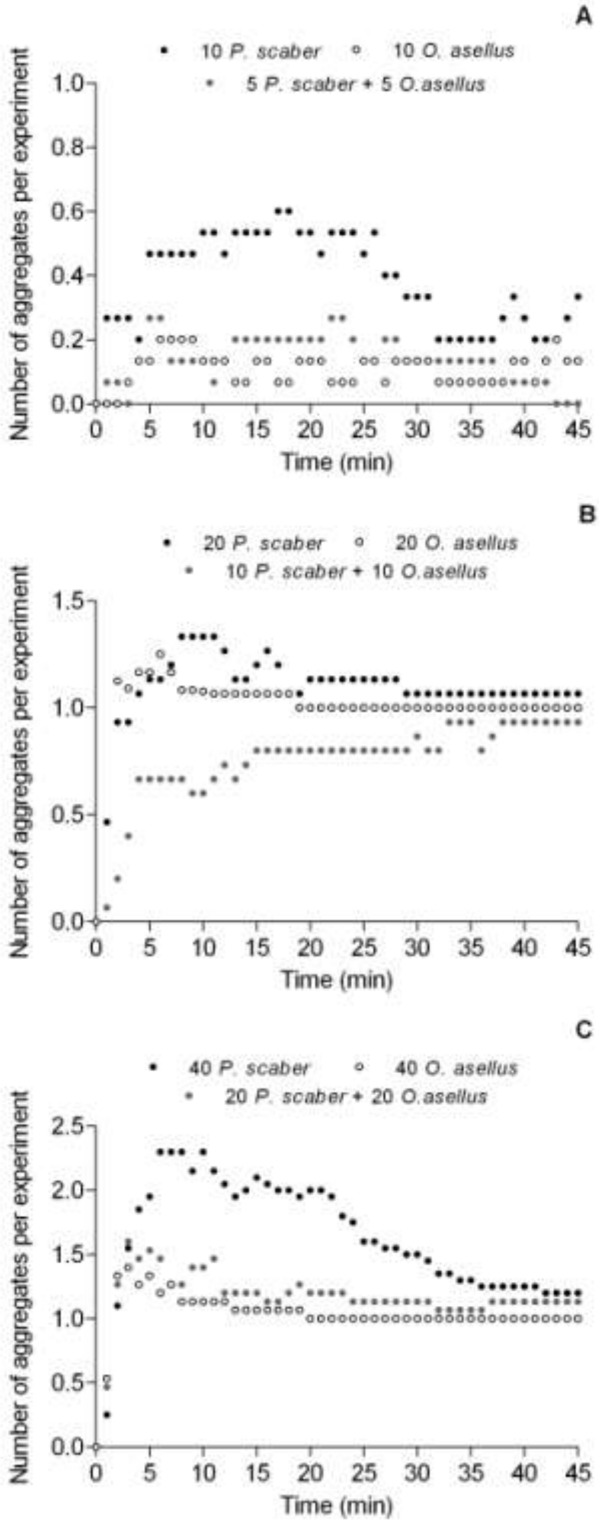


**Figure S3.** Dynamics of aggregation (mean number of aggregates formed per experiment) of mono- and heterospecific groups in experiments with 10 individuals (A), 20 individuals (B) and 40 individuals (C). There are no difference between the final number of clusters formed in experiments with 20 individuals (Kruskal-Wallis test, KW= 2.933, p= 0.2307) and 40 individuals (Kruskal-Wallis test, KW= 3.217, p= 0.2002).
